# Ixabepilone: Overview of Effectiveness, Safety, and Tolerability in Metastatic Breast Cancer

**DOI:** 10.3389/fonc.2021.617874

**Published:** 2021-07-06

**Authors:** Nuhad K. Ibrahim

**Affiliations:** Department of Breast Medical Oncology, Division of Cancer Medicine, The University of Texas MD Anderson Cancer Center, Houston, TX, United States

**Keywords:** breast cancer, cancer management, clinical management, chemotherapy, women’s cancer, drug resistance, ixabepilone

## Abstract

Treatment algorithms for metastatic breast cancer describe sequential treatment with chemotherapy and, if appropriate, targeted therapy for as long as the patient receives benefit. The epothilone ixabepilone is a microtubule stabilizer approved as a monotherapy and in combination with capecitabine for the treatment of metastatic breast cancer in patients with demonstrated resistance to anthracyclines and taxanes. While chemotherapy and endocrine therapy form the backbone of treatment for metastatic breast cancer, the epothilone drug class has distinguished itself for efficacy and safety among patients with disease progression during treatment with chemotherapy. In phase III trials, ixabepilone has extended progression-free survival and increased overall response rates, with a manageable toxicity profile. Recent analyses of subpopulations within large pooled datasets have characterized the clinical benefit for progression-free survival and overall survival for ixabepilone in special populations, such as patients with triple-negative breast cancer or those who relapsed within 12 months of prior treatment. Additional investigation settings for ixabepilone therapy discussed here include adjuvant therapy, weekly dosing schedules, and ixabepilone in new combinations of treatment. As with other microtubule stabilizers, ixabepilone treatment can lead to peripheral neuropathy, but evidence-based management strategies may reverse these symptoms. Dose reductions did not appear to have an impact on the efficacy of ixabepilone plus capecitabine. Incorporation of ixabepilone into individualized treatment plans can extend progression-free survival in a patient population that continues to represent an unmet need.

## Introduction

Breast cancer (BC) is the most common malignancy that affects women in the United States, occurring in ~276,000 women nationwide (1). More than 40,000 US deaths are associated with BC annually (1). In 2013, approximately 138,000 women lived with metastatic BC (mBC) in the United States, of whom 72% had disease progression from an initial diagnosis of Stage I-III disease and 28% were diagnosed with Stage IV disease (2).

Treatment selection for Stage IV BC is guided by tumor expression of targetable receptors, including the estrogen receptor (ER), progesterone receptor (PR), and the human epidermal growth factor receptor 2 (HER2) (3, 4). The recommended first-line treatment for patients with hormone receptor–positive, HER2-negative Stage IV disease is an aromatase inhibitor in combination with a cyclin-dependent kinase (CDK)4/6 inhibitor, a selective ER downregulator in combination with a non-steroidal aromatase inhibitor, or fulvestrant in combination with a CDK4/6 inhibitor (4). Patients with HER2 positive breast cancer receive chemotherapy (taxane) in combination with anti-HER2 therapy (trastuzumab +/- pertuzumab). The addition of an anti-HER agent to an anti-estrogen agent may be employed in situations where the use of cytotoxic chemotherapy is not further advisable due to tolerance or chemoresistance issues. Without a known receptor for targeted therapy, guidelines for advanced metastatic, triple-negative breast cancer (TNBC; ER negative, PR negative, HER2 negative) recommend sequential therapy with chemotherapy with or without targeted therapy, whenever indicated, for as long as there is patient benefit (3, 4). Patients with recurrent mBC of any subtype are transitioned to regimens that include chemotherapy, with or without agents targeted for these receptors (4). No chemotherapeutic agent has demonstrated higher efficacy when compared with other single-agent regimens in the first-line setting, and so treatments are selected with consideration of safety profile and quality of life (3). Although current treatments are not curative for advanced mBC, they can provide palliative care and extend progression-free survival (PFS) (3). Chemotherapy is associated with longer overall survival (OS) compared with placebo; however, clinical trials with targeted agents have not demonstrated an OS benefit with newer agents, in part due to crossover and tumor subtype heterogeneity (3).

Despite the treatment selection for mBC, the tumor tends to develop resistance and disease progression occurs, requiring repeated change of the treatment regimen. The frequent exposure to chemotherapy throughout BC treatment—in particular, taxane-based regimens (e.g., paclitaxel, docetaxel) in the adjuvant setting—places evolutionary pressure on tumor cells to acquire genetic and non-genetic properties that evade drug activity (5). Resistance can be genetically encoded during tumorigenesis, which is typically considered primary resistance, or can be acquired through selection of cancer cells that do not die during the initial phases of treatment, also known as acquired or secondary resistance (6). Tumor resistance to therapy occurs through the selective upregulation of survival pathways and/or the downregulation of cell death pathways (7). In actively proliferating tumor cells, the stabilization of the tumor cell cytoskeleton disrupts the cell cycle, and permits activation of apoptotic pathways (5, 8, 9). In addition, protein phosphorylation or changes in gene expression can underlie drug resistance in cancer (10). Identifying the most efficacious sequence of antitumor agents in the presence of drug resistance remains an unmet need in mBC.

Ixabepilone is a semi-synthetic analog of epothilone B with microtubule inhibitory activity that is approved for use in combination with capecitabine for the treatment of metastatic or locally advanced BC after failure of an anthracycline and a taxane. Ixabepilone is also approved for use as a monotherapy for the treatment of metastatic or locally advanced BC in patients after failure of an anthracycline, a taxane, and capecitabine. Ixabepilone has displayed properties that enable it to evade common resistance mechanisms, and may represent a drug of choice for patients with recurrent disease. The ixabepilone every 3-week dosing schedule may be preferred for patients who desire to reduce visits to the infusion center during periods of social distancing, as experienced during the COVID-19 pandemic, or who have other logistical challenges (11).

## Resistance to Microtubule Inhibitors in Breast Cancer

Cancer cells can overcome drug-induced therapeutic stress with several mechanisms, including, but not limited to: 1) expression of adenosine triphosphate (ATP)-binding cassette (ABC) proteins that confer a multidrug resistance (MDR) phenotype; 2) reduced susceptibility to apoptosis; 3) mutations in drug targets; 4) changes in cell cycle; 5) alterations in drug metabolism (6). Within the superfamily of ABC transporters, three efflux pumps are known to have individual binding profiles with overlapping specificities (12). P-glycoprotein (P-gp), also known as MDR1, and multidrug resistance protein (MRP) can facilitate resistance to anthracycline and taxanes, and these proteins have been observed in mBC and ovarian cancer (13). Taxane resistance has also been linked to mutations of β-tubulin, the protein at the growing end of the microtubule (12). These mechanisms may develop heterogeneously in the tumor or between metastases, generating a disease that responds differently to treatments (10). For capecitabine and nucleobase/nucleoside analogs that disrupt DNA replication, clinical resistance may reflect the proportion of cells in the S phase of the cell cycle within the tumor, which creates a limited therapeutic window (14). However, higher dosages of these drugs or other strategies to extend the therapeutic window are often associated with unacceptable toxicity (14).

Ixabepilone is a microtubule-stabilizing agent that binds directly to β-tubulin subunits and suppresses their dynamic instability, blocking the mitotic phase of the cell division cycle and inducing cell death (15). Differences in molecular structure between antimicrotubule drug classes create different pharmacokinetic and pharmacodynamic profiles, which affect treatment efficacy and usage (12). Although both taxanes and epothilones are microtubule stabilizers, they are structurally unrelated and have different β-tubulin binding modes (16). In preclinical studies, ixabepilone has demonstrated activity in taxane-resistant cell lines, despite the presence of ABC efflux drug pumps and β-tubulin mutations (17). Compared with taxanes, epothilones have a higher affinity for β-tubulin and are not substrates of P-gp, which permits ixabepilone to maintain activity against tumor cells that are resistant to taxanes and/or anthracyclines through upregulation of P-gp expression (6, 18). Early evidence of antitumor activity in a range of xenograft models has supported the study of ixabepilone in breast cancer as well as in ovarian cancer (18).

## Monotherapy With Ixabepilone in mBC

The efficacy and safety of ixabepilone 40 mg/m^2^ administered intravenously (IV) every 3 weeks was demonstrated in Study 081, a phase II, single-arm, multicenter trial conducted in women with mBC or locally advanced BC with resistance to anthracyclines, taxanes, and capecitabine (19). In this heavily pretreated study population, participants had received up to five previous chemotherapy-based regimens, including less than or equal to three courses in the metastatic setting. Study 081 enrolled 126 participants, of whom 75% had received prior chemotherapy, and 48% had received three chemotherapy regimens for metastatic disease. Baseline demographics and disease characteristics are shown in **Table 1**. The independently assessed overall response rate (ORR) was 11.5% (95% confidence interval [CI], 6.3-18.9%; **Table 2**) overall and 12% among participants with TNBC. The median duration of response was 5.7 months (95% CI, 4.4-7.3 months), and 13% of participants had a best response of stable disease for >6 months. The durable response of ixabepilone monotherapy among patients with extensive prior treatment supported the marketing approval of ixabepilone as monotherapy for the treatment of metastatic or locally advanced breast cancer in patients after failure of an anthracycline, a taxane, and capecitabine (34). In addition, these data support a role for ixabepilone in participants with tumors resistant to microtubule-targeted taxanes.

**Table 1:** Baseline characteristics across clinical trials.

	Study 081 (19)	CA163-046 (20, 21)		CA163-048 (22)		TITAN (23)		UNICANCER-PACS08 (24)	
	Ixabepilone (n=126)	Ixabepilone + Capecitabine (n=375)	Capecitabine (n=377)	Ixabepilone + Capecitabine (n=609)	Capecitabine (n=612)	Ixabepilone arm (n=306)	Paclitaxel arm (n=308)	Ixabepilone arm (n=364)	Docetaxel arm (n=398)
**Age, median (range)**	51	53	52	53	53	53	56	53	53.5
	(30-78)	(25-76)	(25-79)	(23-78)	(24-81)	(22-80)	(22-85)	(26-71)	(24-71)
**Baseline performance status**	KPS, n (%)	KPS, n (%)		KPS, n (%)		ECOG PS, n (%)		ECOG PS, n (%)	
	100 33 (26)	90-100 253 (67)	90-100 237 (63)	90-100 406 (67)	90-100 453 (74)	0 266 (87)	0 270 (88)	0 277 (90)	0 311 (90)
	80-90 88 (70)	70-80 119 (32)		70-80 195 (32)	70-80 156 (25)	1 33 (11)	1 35 (11)	1 31 (10)	1 35 (10)
	<80 5 (4)		70-80 136 (36)	<70 2 (<1)	<70 2 (<1)				
**Receptor status**	n (%)	n (%)		n (%)		n (%)		n (%)	
	Hormone receptor status	Hormone receptor status^†^	Hormone receptor status^†^	ER status	ER status	TNBC status	TNBC status	TNBC	TNBC
				ER+ 341 (56)	ER+ 330 (54)		TNBC 308 (100)	N0 153 (42)	N0 151 (38)
				ER- 226 (37)	ER- 250 (41)	TNBC 306 (100)		N+ 126 (35)	N+ 155 (39)
	Positive 65 (52)	Positive 177 (47)	Positive 184 (49)	HER2 status	HER2 status			ER+/PR-/HER2-	ER+/PR-/HER2-
	Negative 51 (40)	HER2 status		HER2+ 85 (14)	HER2+ 100 (16)			N+ 82 (23)	N+ 83 (21)
	HER2 status	HER2+ 59 (16)	HER2 status	HER2- 396 (65)	HER2- 396 (65)				
	HER2+ 9 (7)	TNBC status	HER2+ 53 (14)	TNBC status	TNBC status				
	HER2- 91 (72)	TNBC 91 (24)		TNBC 122 (20)	TNBC 134 (22)				
	TNBC status		TNBC status						
	TNBC 42 (33)		TNBC 96 (26)						
**Extent of disease**	Number of disease sites	Number of disease sites		Number of disease sites		Primary tumor stage, n (%)		Primary tumor stage, n (%)	
	1-2 45 (36)	≥2 332 (89)	≥2 341 (90)	≥2 422 (70)	≥2 427 (70)	T1 137 (45)	T1 141 (46)	pT1 130 (36)	pT1 131 (33)
	3-4 62 (49)	<2 43 (11)	<2 36 (10)	<2 184 (30)	<2 185 (30)	T2 148 (48)	T2 158 (51)	pT2 211 (58)	
	≥5 19 (15)					T3 21 (7)	T3 9 (3)	pT3 23 (6)	pT2 247 (62)
								pT4 0 (0)	pT3 19 (5)
									pT4 1 (<1)
	Visceral disease	Site of visceral disease		Site of visceral disease		Primary nodal stage, n (%)		Primary nodal stage, n (%)	
	Liver and/or lung 97 (77)	Liver 245 (65)	Liver 228 (61)	Liver 273 (45)	Liver 276 (45)	N0 205 (67)	N0 208 (68)	N0 153 (42)	N0 153 (38)
				Lung 221 (36)	Lung 217 (35)		N+ 100 (32)	N+ 211 (58)	N+ 244 (61)
						N+ 101 (33)			
		Lung 180 (48)	Lung 174 (46)						

ECOG, Eastern Cooperative Oncology Group; ER, estrogen receptor; HER2, human epidermal growth factor receptor; KPS, Karnofsky Performance Status; N, node status; PR, progesterone receptor; PS, performance status; T, tumor stage; TNBC, triple-negative breast cancer; +/-, positive/negative.

†Hormone receptor positive=ER+ and/or PR+.

**Table 2:** Summary of key efficacy measures across the clinical studies with ixabepilone in breast cancer.

Phase	Patients, n	Population	Treatment	Efficacy	Study (year)	Ref
**Ixabepilone monotherapy**						
II	65	Patients with mBC previously treated with adjuvant anthracycline	Ixabepilone 40 mg/m^2^ IV Q3W	ORR: 41.5% (95% CI, 29.4-54.4%); mTTP: 4.8 mo; mOS: 22.0 mo	Roché et al.	(25)
II	126	Patients with mBC who progressed during treatment with an anthracycline, taxane, and capecitabine	Ixabepilone 40 mg/m^2^ IV Q3W	ORR (IRF): 11.5% (95% CI, 6.3-18.9%), ORR (INV): 18.3%; mPFS: 3.1 mo; mOS: 8.6 mo; mDoR: 5.7 mo	Perez et al.	(19)
II	49	Patients with mBC who progressed during or within 4 mo of taxane therapy	Ixabepilone 40 mg/m^2^ or 50 mg/m^2^ IV Q3W	ORR (INV): 12% (95% CI, 4.7-26.5%); mDoR: 10.4 mo; mTTP: 2.2 mo; mOS: 7.9 mo	Thomas et al.	(26)
II	52	Patients with mBC resistant to taxanes and previously treated with anthracyclines	Ixabepilone 40 mg/m^2^ IV Q3W	ORR (IRF): 11.5% (95% CI 4.4-23.4%); mDoR: 3.6 mo; mTTP: 2.8 mo; mOS: 12.4 mo	Aogi et al.	(27)
**Ixabepilone in combination with capecitabine**						
II	103	Patients with HER2-negative disease	Ixabepilone 20 mg/m^2^ IV + carboplatin (area under the curve 2.5) IV on days 1 and 8 of a 21-day cycle	ORR: 30.4% in TNBC and 34% in hormone receptor–positive HER2-negative disease; mPFS: 7.6 mo in TNBC and 7.6 mo in hormone receptor–positive HER2-negative disease; mOS: 12.5 mo in TNBC and 17.9 mo in hormone receptor–positive HER2-negative disease	Osborne et al.	(28)
III	752	Patients with locally advanced BC or mBC previously treated with or resistant to anthracyclines and resistant to taxanes	Ixabepilone 40 mg/m^2^ IV Q3W + capecitabine 2000 mg/m^2^ on days 1-14 of a 21-day cycle	mPFS: 5.8 mo (95% CI, 5.45-6.97) *vs* 4.2 mo (95% CI, 3.81-4.50; HR, 0.75; *P*=0.0003); ORR (IRF): 35% *vs* 14% (OR, 3.2; *P*<0.0001); mDoR: 6.4 mo *vs* 5.6 mo; mOS: 12.9 mo *vs* 11.1 mo	Thomas et al.; Hortobagyi et al.	(20, 21)
III	1221	Women with locally advanced BC or mBC previously treated with anthracyclines and taxanes	Ixabepilone 40 mg/m^2^ IV Q3W + capecitabine 2000 mg/m^2^ on days 1-14 of a 21-day cycle	Unadjusted mOS: 16.4 mo *vs* 15.6 mo (HR, 0.9; *P*=0.1162); prespecified adjusted Cox regression of mOS: HR, 0.85; 95% CI, 0.75 to 0.98; *P*=0.0231) mPFS: 6.24 mo *vs* 4.4 mo (HR, 0.79; *P*=0.0005); ORR (INV): 43% *vs* 29% (*P*<0.0001); DoR: 6.1 mo *vs* 6.3 mo	Sparano et al.	(22)
Meta-analysis	2637 (OS)	OS: 3 studies (CA163-046, CA163-068; pooled dataset)	Ixabepilone 40 mg/m^2^ IV Q3W + capecitabine 2000 mg/m^2^ on days 1-14 of a 21-day cycle	OS HR, 0.91 (95% CI, 0.84-0.99)	Li et al.	(29)
				PFS HR, 0.79 (95% CI, 0.74-0.85)		
	3387 (PFS, ORR)	PFS, ORR: 4 studies (CA163-046, CA163-068; pooled dataset [<65 y and ≥65 y])		ORR RR, 1.77 (95% CI, 1.45-2.15)		
**Ixabepilone as adjuvant treatment**						
III	762	Patients with resectable, non-metastatic, poor-prognosis BC	3x FEC100 Q3W + 3x ixabepilone 40 mg/m^2^ IV Q3W, or 3x FEC100 Q3W + 3x docetaxel 100 mg/m^2^ Q3W	5-y DFS rate: 83% (95% CI, 79-87%) *vs* 79% (95% CI, 75-83%; HR, 0.80; *P*=0.175); 5-y OS rate: 88% *vs* 87% (HR, 0.97; *P*=0.897)	Campone et al.	(24)
III	614	Patients with operable, previously untreated TNBC	4x doxorubicin 60 mg/m^2^ + cyclophosphamide 600 mg/m^2^ Q3W, followed by 4x ixabepilone 40 mg/m^2^ Q3W, or 12x paclitaxel 80 mg/m^2^ every week	3-y DFS rate: 88.6% *vs* 88.8% (not significant); 5-y DFS rate: 87.1% *vs* 84.7% (not significant); 3-y OS rate: 92.4% *vs* 93.8% (not significant); 5-y OS rate: 89.7% *vs* 89.6% (not significant)	Yardley et al.	(23)
**Subset analyses of special patient populations**						
Pooled phase III	1973	Participants with KPS 70%-80% and KPS 90%-100% in 2 phase III trials	Ixabepilone 40 mg/m^2^ IV Q3W in combination + capecitabine 2000 mg/m^2^ on days 1-14 of a 21-day cycle	KPS 70%-80%: mPFS: 4.6 mo (95% CI, 4.2-5.6) *vs* 3.1 mo (95% CI, 2.7-3.9; HR, 0.76; *P*=0.0021); mOS: 12.3 mo *vs* 9.5 mo (HR, 0.75; *P*=0.0015) KPS 90%-100%: mPFS: 6.0 (95% CI, 5.6-6.6) *vs* 4.4 (95% CI, 4.2-5.3; HR, 0.82; *P*=0.0009); mOS: 16.7 mo *vs* 16.2 mo (*P*=0.8111)	Roché et al.	(30)
Pooled phase III	293	Participants with PARR disease in 2 phase III trials	Ixabepilone 40 mg/m^2^ IV Q3W in combination + capecitabine 2000 mg/m^2^ on days 1-14 of a 21-day cycle	PARR: mPFS: 5.6 mo (95% CI, 4.6-6.9) *vs* 2.8 (95% CI, 2.2-3.7; HR, 0.58; *P*<0.0001); mOS: 15.1 mo *vs* 12.5 mo (HR, 0.84; *P*<0.2081)	Jassem et al.	(31)
Pooled phase III	251 (≥65 y) 1721 (<65 y)	Participants aged <65 y and ≥65 y in 2 phase III trials	Ixabepilone 40 mg/m^2^ IV Q3W in combination + capecitabine 2000 mg/m^2^ on days 1-14 of a 21-day cycle	Age ≥65 y: mPFS: 5.5 mo *vs* 3.9 mo (HR, 0.77; 95% CI, 0.59-1.02); mOS 13.9 mo *vs* 12.2 mo (HR, 1.07; 95% CI, 0.81-1.40) Age <65 y: mPFS 5.6 mo *vs* 4.2 mo (HR, 0.81; 95% CI, 0.73-0.90); mOS: 14.7 mo *vs* 13.9 mo (HR, 0.90; 95% CI, 0.81-1.01)	Vahdat et al.	(32)
Pooled phase III	443	Participants with TNBC in 2 phase III trials	Ixabepilone 40 mg/m^2^ IV Q3W in combination + capecitabine 2000 mg/m^2^ on days 1-14 of a 21-day cycle	TNBC: mPFS: 4.2 mo (95% CI, 3.6-4.4) *vs* 1.7 mo (95% CI, 1.5-2.4; HR, 0.64; *P*<0.0001); mOS 10.4 mo *vs* 9.0 mo (HR, 0.88; *P*=0.1802)	Rugo et al.	(33)

BC, breast cancer; CI, confidence interval; DFS, disease-free survival; DoR, duration of response; FEC, 5-FU+epirubicin+cyclophosphamide; HER2, human epidermal growth factor receptor; HR, hazard ratio; INV, investigator-assessed; IRF, independent radiology facility or committee; IV, intravenous; KPS, Karnofsky Performance Status; mBC, metastatic breast cancer; mDoR, median DoR; mo, month; mOS, median OS; mPFS, median PFS; mTTP, median time to progression; OR, odds ratio; ORR, objective response rate; OS, overall survival; PARR, post-adjuvant rapidly relapsing; PFS, progression-free survival; Q3W, every 3 weeks; TNBC, triple-negative breast cancer; y, year.

## Ixabepilone in Combination With Capecitabine

Clinical trials have shown that there is a synergistic antitumor effect of ixabepilone in combination with capecitabine (15). The open-label, randomized, active-controlled, multi-national phase III CA163-046 study evaluated the safety and efficacy of ixabepilone in combination with capecitabine compared with capecitabine monotherapy in women with anthracycline/taxane-pretreated mBC that was resistant to anthracyclines and taxanes (20, 21). This trial enrolled patients with mBC who were previously treated with anthracycline as one of less than or equal to three prior chemotherapy regimens and who had met predefined criteria for tumor resistance to anthracyclines and taxanes (20). Seven hundred fifty-two patients were randomized 1:1 to receive either ixabepilone 40 mg/m^2^ IV every 3 weeks in combination with capecitabine (I+C) 1000 mg/m^2^ orally twice daily on days 1-14 of a 21-day cycle or capecitabine (C) 1250 mg/m^2^ orally twice daily on days 1-14 of a 21-day cycle (21).

The trial met its primary endpoint with a significant difference in median PFS: 5.8 months in the I+C arm compared with 4.2 months in the C arm (hazard ratio [HR], 0.75; 95% CI, 0.64-0.88; stratified P=0.0003) (20). Participants who received I+C had significantly higher rates of tumor response and a lower risk of progression (20, 21). Exploratory subset analysis demonstrated a PFS benefit in most subpopulations of participants defined by baseline characteristics, including Karnofsky Performance Status (KPS), prior chemotherapy, and TNBC tumors (20). Notable exceptions to having significantly longer median PFS associated with I+C treatment compared with C were ER-positive tumors, HER2-positive tumors, and lack of resistance to anthracyclines (20). These data supported the approval of ixabepilone in combination with capecitabine in the United States in 2007 for the treatment of metastatic or locally advanced breast cancer in patients after failure of an anthracycline and a taxane.

Median OS was not significantly different between the I+C and C study arms (12.9 months *vs* 11.1 months; HR, 0.90; 95% CI, 0.77-1.05) (21). Among the predefined subset analyses, there was a significant difference in OS between treatment arms among the subpopulation with KPS 70%-80%; the median OS was 10.1 months and 7.8 months in the I+C (n=119) and C (n=136) arms, respectively (HR, 0.75; 95% CI, 0.58-0.98). No other subgroups had significant differences in OS between study arms.

Most trials in mBC have not shown an OS benefit for several reasons: varied treatment strategies before and after study participation, underpowered trial populations, broad inclusion criteria, and limited follow-up (3, 21, 35). Preplanned or exploratory subset analyses can be a good start to addressing these questions, but these calculations are limited by sample size. Whether I+C extended OS compared with C was addressed in study CA163-048 (22). With enrollment criteria and study design similar to CA163-046, study CA163-048 was designed and powered to assess OS as a primary endpoint, with a larger enrollment and prespecified covariates to control for known prognostic factors (20–22). The study evaluated the safety and efficacy of I+C compared with C in women with mBC with less than or equal to two chemotherapy regimens, including anthracycline- and taxane-containing regimens (22). Additionally, participants in CA163-048 were not required to be resistant to anthracyclines or taxanes, or to have received previous treatment for metastatic disease.

As with study CA163-046, the treatments consisted of ixabepilone 40 mg/m2 IV every 3 weeks in combination with capecitabine orally twice daily 1000 mg/m^2^ on days 1-14 of a 21-day cycle (I+C) and capecitabine 1250 mg/m2 orally twice daily on days 1-14 of a 21-day cycle (C) (22). The study assigned 609 participants to the I+C arm and 612 to the C arm (N=1221) (22). Slightly more participants in the I+C arm had a KPS of 70%-80% compared with the C arm (32% *vs* 25%, respectively), but otherwise the baseline characteristics were similar between the treatment arms.

The CA163-048 trial did not meet its primary endpoint of OS as evaluated with an unadjusted Cox proportional hazards model (16.4 months in I+C *vs* 15.6 months in C; HR, 0.9; 95% CI, 0.78-1.03; P=0.1162) (22). However, the preplanned adjusted Cox regression demonstrated a significant difference in OS when controlled for age, KPS, ER status, visceral disease, and other prespecified covariates (HR, 0.85; 95% CI, 0.75-0.98; P=0.0231). As in CA163-046, a subanalysis of participants with KPS 70%-80% showed longer OS with I+C compared with C: 14.0 *vs* 11.3 months, respectively (HR, 0.76; 95% CI, 0.60-0.96). Participants receiving I+C had a significantly greater PFS benefit compared with those receiving C: 6.24 months *vs* 4.4 months (HR, 0.79; 95% CI, 0.69-0.90; P=0.0005), and the ORR was significantly higher in the I+C arm compared with the C arm: 43% (95% CI, 39-48%) *vs* 29% (95% CI, 25-33%), P<0.0001.

These results were confirmed with an independent meta-analysis of OS data from three studies comparing I+C and C (29). The majority of data included in this meta-analysis were from CA163-046 and CA163-048, and there was no significant heterogeneity between study trials. Li et al. performed a systematic review and meta-analysis (n=5247) that showed a significant difference in OS between participants receiving I+C and C (HR, 0.91; 95% CI, 0.84-0.99; P=0.03). As expected, PFS and ORR were also significantly longer (HR, 0.79; 95% CI, 0.74-0.85) and higher (relative risk, 1.77; 95% CI, 1.45-2.15), respectively, for I+C *vs* C, although there was heterogeneity within the ORR dataset.

## Efficacy of Ixabepilone in Special Populations

The similarity of study designs and enrollment criteria for CA163-046 and CA163-048 permitted pooling of individual patient data for efficacy analyses (31). The pooled study population explored the efficacy of I+C compared with C across subpopulations defined by KPS, age, post-adjuvant rapidly relapsing (PARR) disease, and TNBC (30–33). Key efficacy measures for these subgroup analyses are presented in **Table 2**.

### Performance Status and Efficacy Outcomes

Roché et al. performed a subset analysis of efficacy and safety of I+C in groups defined by performance status (KPS 70%-80% or 90%-100%) within the pooled dataset (30). Among participants with KPS 70%-80%, there was significantly longer PFS and OS in the I+C arm compared with the C arm (PFS: 4.6 *vs* 3.1 months, respectively [HR, 0.76, P=0.0021]; OS, 12.3 *vs* 9.5 months, respectively [HR, 0.75; P=0.0015]). The ORR was higher in the I+C arm compared with the C arm (32% *vs* 19%, respectively). Within the KPS 70%-80% subpopulation, I+C was associated with longer PFS compared with C in participants previously treated with an anthracycline or taxane (5.6 *vs* 3.9 months, respectively; HR, 0.74; 95% CI, 0.58-0.95), and longer OS regardless of history of treatment with anthracycline or taxane (pretreated: 14.0 *vs* 11.3 months, respectively [HR, 0.76; 95% CI, 0.60-0.96]; resistant: 10.1 *vs* 7.8 months, respectively [HR, 0.75; 95% CI, 0.58-0.98]). Similarly, participants with KPS 90%-100% received greater PFS and ORR benefit from treatment with I+C compared with the C arm. However, the difference in OS between treatment arms was not significant, with a median OS of 16.7 months in the I+C treatment arm and 16.2 months in the C arm (HR, 0.98; 95% CI, 0.87-1.12; P=0.8111).

### Efficacy Outcomes in Elderly Patients

The efficacy and safety of I+C was compared with C monotherapy in subpopulations defined by age (<65 and ≥65 years) from the pooled analysis of studies CA163-046 and CA163-048 (32). Vahdat et al. found that I+C was associated with numerically longer median PFS compared with C in participants ≥65 years (5.5 *vs* 3.9 mo; HR, 0.77; 95% CI, 0.59-1.02); the CI of this HR extended beyond 1.0, perhaps due to the smaller sample size (32). Similarly, Sparano et al. found significantly longer PFS among participants ≥50 years who received I+C compared with C in CA163-048 (HR, 0.80; 95% CI, 0.68-0.94) (22).

### Clinical Outcomes in Patients With Early Relapse With Adjuvant Anthracyclines and Taxanes

Jassem et al. characterized efficacy and safety among participants who were resistant to taxane and anthracyclines with disease recurrence within 12 months of adjuvant or neoadjuvant therapy in the pooled phase III trial data (31). PARR is associated with poor prognosis and may reflect inherent drug resistance. There were 293 PARR participants in CA163-046 (n=55, representing 7.3% of the patient population) or CA163-048 (n=238, or 19.5%). Within the pooled PARR subpopulation, a higher proportion of patients had TNBC compared with the overall pooled population (40% *vs* 22%, respectively), but otherwise, the baseline demographics were similar in the subpopulation and the pooled dataset and between treatment arms. The median PFS in participants with PARR disease was 5.6 months in the I+C arm and 2.8 months in the C arm (HR, 0.58; 95% CI, 0.45-0.76; P<0.0001). The investigators also found that tumor response rates were higher in patients who received I+C compared with those who received C (ORR: 46% *vs* 24%, respectively), with complete response occurring in 7% of the I+C arm and 2% of the C arm. There was no significant difference in the median OS between the treatment arms. These data suggest ixabepilone provided benefit to study participants with primary drug resistance.

### Clinical Outcomes in TNBC

An analysis of efficacy and safety of I+C compared with C in patients with TNBC was performed by Rugo et al. (33) Just as with PARR disease, patients with TNBC have poor prognosis and limited treatment options. The pooled analysis included 443 participants with TNBC, of whom 213 received I+C combination treatment and 230 received C. Study participants with TNBC had significantly longer PFS in the I+C arm compared with the C arm: 4.2 months *vs* 1.7 months, respectively (HR, 0.64; 95% CI, 0.52-0.78; P<0.0001). The ORR for participants with TNBC was 31% compared with 15% in the I+C and C arms, but the difference in median OS for the TNBC subset between the arms was not significant.

## Ixabepilone in the Adjuvant Setting

Treatment options for people with TNBC are limited: early clinical responses may still lead to rapid progression and poor prognosis. The significant prolongation of PFS in participants of studies CA163-46 (20) and CA163-68 (22) with TNBC suggested that ixabepilone may provide benefit to women with TNBC at early stages of treatment (20, 22). The phase III TITAN trial evaluated ixabepilone in the adjuvant setting for early stage patients with TNBC (23).

After undergoing locoregional surgery, study participants in TITAN were treated with four cycles of doxorubicin/cyclophosphamide every 3 weeks, followed by either ixabepilone 40 mg/m^2^ every 3 weeks for four cycles or paclitaxel 80 mg/m^2^ once weekly for a 12-week period. TITAN’s primary endpoint was rates of disease-free survival (DFS), which was defined as the time between randomization and the first documented disease recurrence event or death from any cause.

Study groups in the TITAN trial were generally balanced for patient demographics and disease characteristics at baseline, although the paclitaxel arm had a higher proportion of participants ≥50 years of age. With a median follow-up of 48 months, adjuvant ixabepilone was not superior to adjuvant paclitaxel in extending 3- and 5-year rates of DFS and OS. The 3-year DFS rate was 88.6% and 88.8% for the ixabepilone and paclitaxel arms, respectively, and the 5-year DFS rate was 87.1% and 84.7% for the ixabepilone and paclitaxel arms, respectively (HR, 0.92; 95% CI, 0.59-1.42; P=0.70). The relapse rate in TITAN was 9.7%, with similar incidence in both treatment arms (9.5% and 9.9% in ixabepilone and paclitaxel, respectively). The large proportion of participants with very early stage disease and the incomplete recruitment may have limited the sensitivity for small differences in DFS and OS rates in this study.

Similarly, the UNICANCER-PACS08 was a multicenter, open-label, randomized, active-controlled phase III trial of adjuvant ixabepilone 40 mg/m^2^ every 3 weeks vs docetaxel 100 mg/m^2^ every 3 weeks in women with early stage, poor prognosis BC treated with fluorouracil (5-FU), epirubicin, and cyclophosphamide combination therapy (FEC100) (24). Within this study population, there was no statistical difference between the study groups in the primary endpoint of the 5-year DFS rate: 79% (95% CI, 75%-83%) and 83% (95% CI, 79%-87%) in the docetaxel and ixabepilone arms, respectively (HR, 0.80; 95% CI, 0.58-1.10; P=0.175). The 5-year OS rate was similar in both treatment arms. Preplanned subgroup analyses for secondary endpoints demonstrated a numerically lower risk of disease recurrence associated with ixabepilone treatment than with docetaxel treatment and a significantly improved distant metastasis-free survival rate at 5 years (HR, 0.58; 95% CI, 0.37-0.90; P=0.014). The reduced risk of relapse and metastasis in a poor-prognosis population may warrant further investigation.

### Ixabepilone in Combination With Targeted Agents

Combination therapy regimens that include ixabepilone have been evaluated in phase II and retrospective studies across a range of tumor types (36, 37). While promising early data have emerged for some combinations, concerns about tolerability indicate caution is needed for other regimens. The safety and efficacy of ixabepilone in combination with trastuzumab was evaluated in women with HER2-positive mBC in a non-randomized study (38). All 39 participants received ixabepilone IV 40 mg/m^2^ on day 1 of a 21-day cycle, and the first dose of trastuzumab 8 mg/kg was administered on day 1 with subsequent doses of 6 mg/kg given every 21 days. Participants who had not received trastuzumab in the metastatic setting were placed in cohort 1; cohort 2 included participants previously treated with trastuzumab for mBC. The ORR and clinical benefit rate (CBR) were 73% (95% CI, 45%-92%) and 80% (95% CI, 52%-96%), respectively, in cohort 1, and 25% (95% CI, 10%-47%) and 42% (95% CI, 22%-63%), respectively, in cohort 2 (38). Time-to-treatment failure was similar in both cohorts (6.6 months vs 6.2 months) (38). The most common treatment-related adverse events (TRAEs) of any grade were fatigue (82%), sensory neuropathy (82%), and anemia (74%). Grade 3/4 events occurring in >5% of participants were sensory neuropathy (18%), neutropenia (18%), leukopenia (11%), and alkaline phosphatase abnormality (8%). Treatment was discontinued in 53% of cohort 1 participants due to toxicity, and the most common reason for discontinuation in cohort 2 was disease progression (50%) (38).

A phase I/II trial in 83 women with HER2-negative mBC aimed to establish a maximum tolerated dose of ixabepilone and sorafenib during the first phase and to evaluate efficacy and safety of the combination in the second phase (39). The minimum tolerated dose was ixabepilone 32 mg/m^2^ every 21 days with 400 mg sorafenib twice daily; this combination was evaluated in the second phase of the trial (n=76). The ORR and CBR in the second phase of the trial were 37% and 43%, respectively, and the median PFS and OS were 4.8 months (95% CI, 3.5-6.3) and 15.5 months (95% CI, 11-20.6). The incidence of fatigue, nausea, rash, and neuropathy were 71%, 71%, 53%, and 51%, respectively, in the second phase of the trial; grade 3/4 neutropenia or febrile neutropenia occurred in 19 participants. Toxicity was the cause of 22% of treatment discontinuations, and 13 participants required hospitalization because of TRAEs recorded in the trial. The investigators concluded that this regimen had unacceptable tolerability for the efficacy benefit.

The combination of ixabepilone with cetuximab was evaluated in women with locally advanced or metastatic TNBC (40). Participants in this open-label, randomized trial received either ixabepilone 40 mg/m^2^ every 21 days as monotherapy (n=40) or ixabepilone 40 mg/m^2^ every 21 days with cetuximab once weekly (400 mg/m^2^ as the loading dose, 250 mg/m^2^ thereafter; n=37). The ORR was 30% (95% CI, 17%-47%) and 36% (95% CI, 21%-53%) in the monotherapy and combination arms, respectively. Median PFS was 4.1 months with each treatment. The incidence of peripheral sensory neuropathy and neutropenia at any grade was 43% and 45%, respectively, in the monotherapy arm and 46% and 54%, respectively, in the combination arm. Cutaneous TRAEs occurred more frequently in the combination arm than in the ixabepilone monotherapy arm. Toxicity-related treatment discontinuations occurred in 20% of participants receiving ixabepilone monotherapy and 35% of participants receiving ixabepilone with cetuximab.

### Ixabepilone in Combination With Chemotherapeutic Agents

In addition to the approved combination of ixabepilone and capecitabine, combinations of ixabepilone with chemotherapeutic agents have been more promising than combinations with targeted therapy. Osborne et al. conducted a multicenter, open-label study of ixabepilone in combination with carboplatin in women with HER2-negative mBC (28). All participants received 20 mg/m^2^ ixabepilone every 21 days and carboplatin on days 1 and 8 of the 21-day cycle. The study enrolled 49 women with TNBC and 54 women with hormone receptor–positive, HER2-negative mBC; efficacy in these cohorts was analyzed separately. The ORR was 30% in participants with TNBC and 34% in those with hormone receptor–positive, HER2-negative mBC. The median PFS was 7.6 months in both arms, while the median OS was 17.9 months in participants with hormone receptor–positive, HER2-negative disease and 12.5 months in participants with TNBC. In the overall study population, the incidence of grade 3/4 neutropenia was 49% and grade 3/4 neuropathy was 9%. Most participants (58%) required both a dose delay and dose modification in response to toxicities, but the tolerability of the regimen was considered manageable.

## Overview of Safety in Phase III Trials

### TRAEs

In all phase III trials, the majority of TRAEs were mild or moderate in severity (20–24, 30–33). Differences in non-hematologic TRAEs between ixabepilone-containing regimens included higher incidences of peripheral neuropathy (PN), fatigue, and diarrhea (20–24, 30–33). Almost all study participants receiving I+C developed hematologic abnormalities; the incidence of hematologic TRAEs varied in the comparator arms of study CA163-046 (20, 21). Among the patients who received ixabepilone monotherapy, the incidence of hematologic abnormalities was generally lower (19, 23, 24).

**Figure 1:**
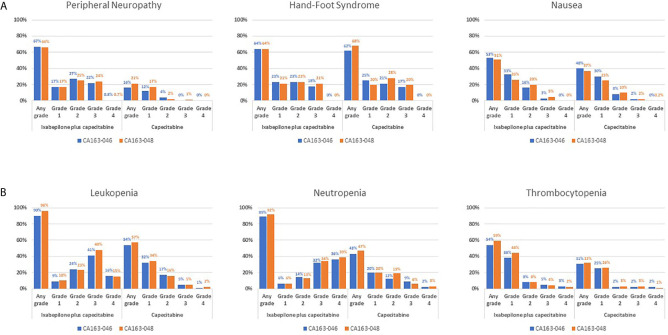
TRAE Incidence Associated With I+C and C Treatment in Pooled Datasets From CA163-046 and CA163-048. Incidence of the most common **(A)** non-hematologic and **(B)** hematologic treatment-related adverse events in the CA163-046 and CA163-048 phase III trials. (20, 22) I, ixabepilone; I+C, ixabepilone plus capecitabine; TRAE, treatment-related adverse event.

**Figure 2:**
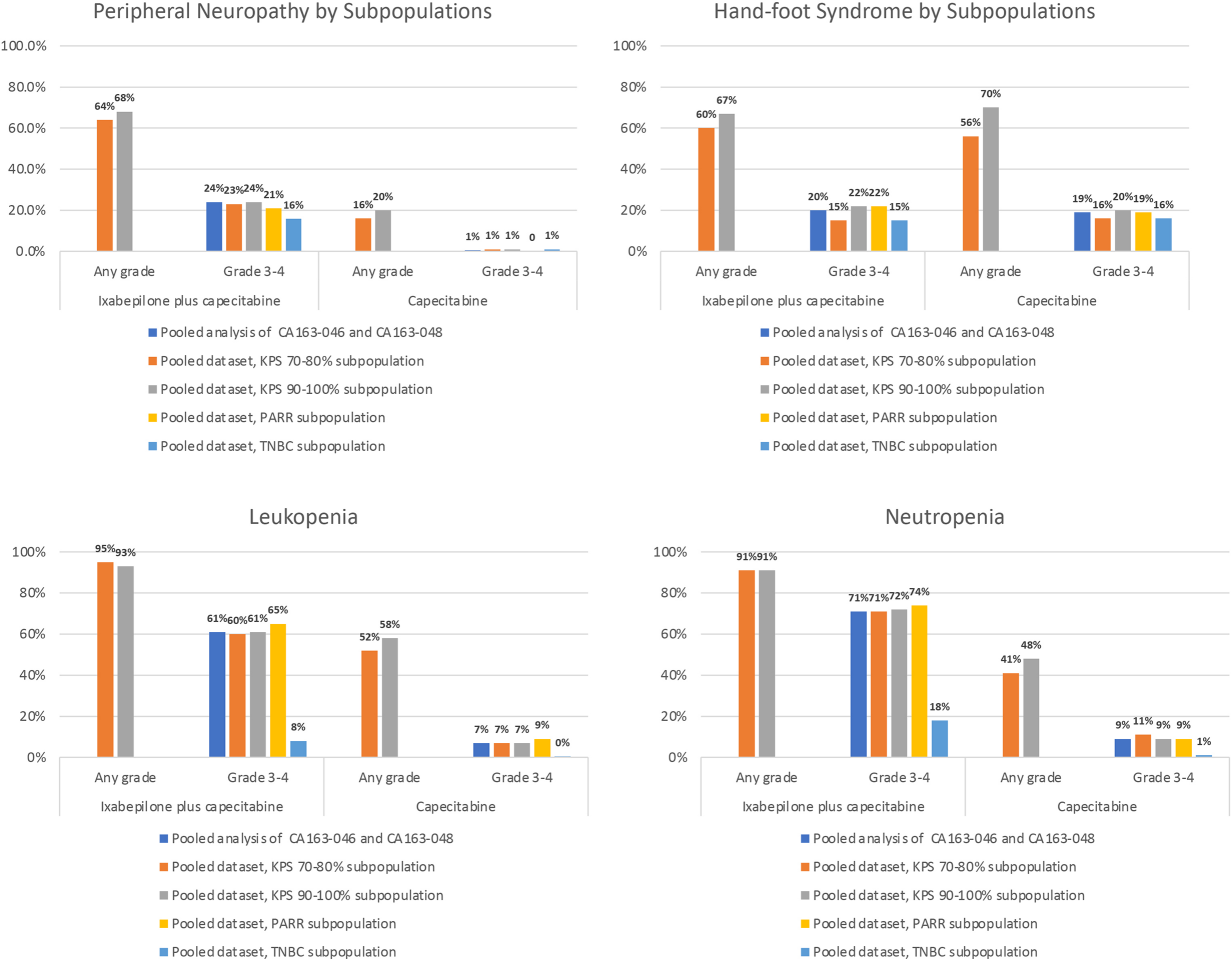
Incidence of the Most Common Non-Hematologic TRAEs Associated With I-C and C in Subpopulations. Incidence of peripheral neuropathy, hand-foot syndrome, leukopenia, and neutropenia in subpopulations of the pooled analysis of CA163-046 and CA163-048 (30, 31, 33). I, ixabepilone; I+C, ixabepilone plus capecitabine; KPS, Karnofsky Performance Status; PPAR, post-adjuvant rapidly relapsing; TNBC, triple-negative breast cancer; TRAE, treatment-related adverse event.

Pooled datasets from CA163-046 and CA163-048 described additional trends in TRAE incidence associated with I+C and C treatment (**Figure 1**) (20, 22). In both trials, PN occurred more frequently at moderate severity in combination therapy compared with C alone. The incidence of hand-foot syndrome and nausea were similar, regardless of treatment arm, suggesting that these TRAEs were associated with C. Hematologic TRAEs, including leukopenia, neutropenia, and thrombocytopenia, occurred at higher grades in the I+C arm compared with the C arm. The incidence of the most common non-hematologic TRAEs associated with I+C and C in subpopulations was similar in subpopulations defined by KPS score, PARR status, or TNBC tumor type (**Figure 2**) (20, 22, 30, 31, 33). Differences in study design and patient population limits the comparison of the incidence of TRAEs between the pooled dataset and other phase III trials.

### Dose Modifications and Other Management Strategies

The protocols of CA163-046 and CA163-048 incorporated guidance on dose modifications to address toxicities associated with ixabepilone and/or capecitabine (20, 41). In CA163-046 and CA163-048, the dose of ixabepilone was reduced in 51% and 48% of participants in the I+C arms, respectively, the dose of capecitabine was reduced in 45% and 49% of participants in the I+C arms, respectively, and the dose of capecitabine was reduced in 37% and 43% of participants in the C arm, respectively (20, 22). Reported discontinuation rates attributed to toxicity ranged from 7.5% to 30% in ixabepilone-containing regimens (20, 22, 23). Frequently reported reasons for dose modifications included PN and hematologic toxicities (20, 22–24). The majority of discontinuations were attributed to disease progression (21, 22).

The evaluation of efficacy of reduced dosages of ixabepilone and/or capecitabine was conducted in a subset analysis (41). In the I+C arm, disease outcomes of participants who had dose modifications during the first four courses of treatment were compared. Outcomes of patients who received early dose reductions (n=219) were compared with the outcomes of participants who received at least four courses, but had dose reductions after the first four courses or no dose reductions during the study—i.e., late/no dose reductions (n=347) (41). Baseline characteristics were similar in the two subpopulations; KPS was ≥90% in 69% and 71% of the early dose reduction subpopulation and the no/late dose reduction subpopulation, respectively. The most common reasons for first ixabepilone dose reduction in the early dose reduction and no/late dose reduction arms were hematologic toxicity (36% and 5%, respectively), non-hematologic toxicity (32% and 6%, respectively), and PN (29% and 19%, respectively). Non-hematologic toxicities were attributed to 59% and 40% of capecitabine dose reductions of early and late/no-dose reduction arms, respectively.

Within the pooled dataset, there was no significant difference between median PFS in the early and late/no dose reduction subpopulations; the median PFS was 7.2 months (95% CI, 6.6-8.0) and 7.0 months (95% CI, 6.5-7.5) among participants with early and late/no dose reductions, respectively (HR, 0.98; 95% CI, 0.83-1.17) (41). The ORR also was similar in these subpopulations: 63% *vs* 55% among early and late/no dose reductions, respectively. These data suggest that ixabepilone at reduced doses, in combination with capecitabine, can maintain efficacy.

Although the subgroup analysis performed by Valero et al. did not present the incidence of TRAEs after dose reduction, there were indications that lower dosages of I+C were associated with manageable tolerability (41). The median number of doses was six (range, 1-44) in the I+C arm of the pooled population, seven (range, 4-44) in the early dose reduction subgroup, and seven (range, 4-42) in the late/no dose reduction subgroup. Within the early dose reduction subgroup, 52% had at least 2 dose reductions compared with 20% of the late/no dose reduction subgroup. For the early and late/no dose reduction subgroups, there were similar proportions of participants who discontinued treatment because of disease progression (55% *vs* 51%, respectively) and study drug toxicity (27% *vs* 25%, respectively). These observations suggest that the dose reduction protocol permitted treatment for maximal efficacy in both subpopulations. The efficacy and safety analysis of I+C compared with C in patients with TNBC performed by Rugo et al. (33) also showed that management of toxicity with dose reductions did not impact efficacy.

The question of whether to initiate treatment with a lower dose of ixabepilone remains under investigation. Early phase studies with ixabepilone in mBC tested a higher dose (50 mg/m^2^ IV every 3 weeks) and/or a shorter infusion period (1 hour), but poor tolerability led investigators to treat with ixabepilone 40 mg/m^2^ IV over a 3-hour infusion in subsequent trials (26). To further test the efficacy of lower doses, two phase II trials evaluated more frequent administration of ixabepilone at lower than standard dosing (42, 43). The results have been mixed. Fountzilas et al. reported a randomized, non-comparative phase II trial in patients with mBC who had not previously received chemotherapy in the metastatic setting (42). In this trial, 32 participants received ixabepilone 40 mg/m^2^ every 3 weeks and 32 participants received ixabepilone 20 mg/m^2^ once weekly. The cumulative dose was slightly higher in the participants who received weekly ixabepilone (240 mg/m^2^ and 231 mg/m^2^ in the ixabepilone 40 mg/m^2^ every 3 weeks arm), but a higher proportion of participants discontinued treatment in the weekly ixabepilone arm (84% and 44%, respectively). The primary endpoint ORR was 45% among participants who received ixabepilone 20 mg/m^2^ once weekly and 44% among participants receiving ixabepilone 40 mg/m^2^ every 3 weeks. Median PFS and OS were numerically longer with weekly ixabepilone, but the CIs overlapped: ixabepilone 20 mg/m^2^ once weekly, 12 months (95% CI, 6-28) and mOS not reached (95% CI, 24-not reached); ixabepilone 40 mg/m^2^ every 3 weeks, 9 months (95% CI, 4-14) and 26 months (95%, 13-not reached), respectively. Analysis of severe adverse events indicated that while a weekly dose of ixabepilone was associated with a lower incidence of neutropenia, there was a greater occurrence of severe leukopenia, sensory neuropathy, and fatigue.

A second phase II trial reported by Smith et al. however, reported shorter PFS and ORR with a weekly dosing schedule for ixabepilone 16 mg/m^2^ (43). In this multicenter, open-label, randomized phase II trial, 176 participants were randomized 1:1 to receive either ixabepilone 16 mg/m^2^ as a 1-hour IV infusion on days 1, 8, and 15 of a 28-day cycle or to receive the standard ixabepilone dosing. The 6-month PFS rate was 43% with the standard ixabepilone dosing compared with 29% with the ixabepilone 16 mg/m^2^ in the modified weekly schedule. The median PFS and ORR were 5.3 months (95% CI, 3.8-6.2) and 14% (95% CI, 7-22%), respectively, with standard dosing, compared with 2.9 months (95% CI, 2.7-5.1) and 8% (95% CI, 3-16%), respectively, with the modified weekly schedule. Most TRAEs were grade 1 or 2. The incidence of grades 3 or 4 TRAEs was higher in participants who received ixabepilone 40 mg/m^2^ every 3 weeks than in those who received ixabepilone 16 mg/m^2^ on a modified weekly schedule: 68.5% *vs* 28%, respectively. PN occurred more frequently and at greater severity in the standard dosing arm.

A recent meta-analysis conducted to synthesize the data from these trials and an additional study of weekly administration of ixabepilone found that there was no difference in PFS and OS between every 3 week and once weekly dosing (29). Comparison of ORR data showed, however, that there was significantly higher tumor response in study participants who received ixabepilone every 3 weeks compared with weekly dosing (29). An open question remains as to whether a lower dose of ixabepilone at the current schedule (every 3 weeks) may improve tolerability while maintaining tumor control, with some TRAEs occurring more frequently with every 3 weeks dosing and no difference in the incidence of other TRAEs as found in the meta-analysis. For instance, starting capecitabine at 2000 mg/m^2^/d has been shown to decrease the incidence of TRAEs associated with this antitumor agent, while maintaining efficacy (44). Current guidelines now recommend both doses (4). Additional studies will be necessary to determine whether initiating ixabepilone at a lower dose to achieve better tolerability can maintain efficacy.

### Management of TRAEs

Management protocols for TRAEs are designed to reduce the toxicity of ixabepilone-containing treatments while permitting continued treatment in the presence of hematologic and non-hematologic toxicity (45).

#### Peripheral Neuropathy

PN is a common TRAE that accompanies antimicrotubule therapy, with reported incidence ranging from 31%-67% at any grade and from 5%-24% at grade 3/4 (19, 20, 22, 23, 46, 47). The onset of PN in a head-to-head phase II trial occurred earlier with ixabepilone than with eribulin (median time to onset 12 weeks *vs* 36 weeks, respectively) (47). The maximum time to resolution was shorter in participants who received ixabepilone: 10.1 weeks compared with 48.4 weeks in the eribulin arm (47). The overall incidence of PN, however, was not significantly different in the ixabepilone and eribulin arms (47). A pooled database of participants in phase II and III clinical trials with ixabepilone provided evidence for a cumulative dosing effect on the development of PN (48). Although the mechanism of antimicrotubule agents on peripheral nerves is unknown, its effect presents as axonal abnormalities or a myelin sheath abnormality in sensory, motor, or autonomic nerve systems (49).

PN symptoms can be reversed with dose reductions of ixabepilone (48). Among patients with grade 3/4 neuropathy, ~80% saw resolution of symptoms within the median time of 5.4-6.2 weeks, and ~85% of study participants reported improvement of symptoms to grade 1 severity within a median time of 4.1-4.5 weeks (48). The recommended management protocol is as follows: patients who report symptoms of grade 2 neuropathy lasting ≥7 days and grade 3 neuropathy lasting <7 days should have a dose reduction of 20%, whereas patients with disabling neuropathy or grade 3 neuropathy that lasts ≥7 days should discontinue treatment (15, 45). An additional 20% dose reduction is recommended if there is a recurrence of symptoms (15).

#### Hematologic Abnormalities and Myelosuppression

Treating physicians should monitor patients for hematologic abnormalities, including myelosuppression and neutropenia, with frequent peripheral blood cell counts (15). Neutropenia is reversible after dose modification; however, deaths attributed to neutropenia have occurred in 1.9% of participants with normal hepatic function or mild hepatic impairment treated with I+C in clinical trials (15). Patients with neutrophil counts <500 cells/mm^3^ for ≥7 days, with febrile neutropenia, or with platelet loss (platelets <25,000/mm^3^ or platelets <50,000/mm^3^ with bleeding) should receive a 20% lower dose of ixabepilone (15). Documented neutrophil counts <1500 cells/mm^3^ should lead to discontinuation (15).

#### Other Severe Non-Hematologic Events

Most grade 3 toxicity should be managed with a 20% decrease in the dose of ixabepilone (45). Recurrence of toxicity should prompt a further dose reduction by 20% (15). Patients with grade 3 hand-foot syndrome or transient grade 3 arthralgia, myalgia, or fatigue may continue at the recommended dose (15). Patients with any grade 4 TRAE should discontinue treatment (15).

## Discussion

Ixabepilone is an epothilone-class antimicrotubule agent that extends PFS in a wide range of patient subtypes with heavily pretreated and hard-to-treat tumor types. Ixabepilone is approved for the treatment of metastatic or locally advanced BC as monotherapy or in combination with capecitabine after failure of an anthracycline and a taxane with or without resistance to capecitabine. Recent analysis of pooled individual patient data from phase III trials demonstrated that I+C was associated with higher response rates and longer PFS compared with C in special patient populations. Ixabepilone has been shown to improve response rates and extend PFS in patients with early relapse following neo/adjuvant treatment with anthracyclines and taxanes, in combination with capecitabine in patients with TNBC who have limited treatment options after having failed previous therapy, and in combination with capecitabine for elderly patients and for those with impaired performance status. Ixabepilone has a well-characterized safety profile, with mild-to-moderate PN that can be reversed with dose modification protocols. Patients should be monitored for neutropenia, which can also be managed with dose reductions. The most common TRAEs associated with ixabepilone are sensory neuropathy, fatigue, and neutropenia. Current protocols appear sufficient to maintain treatment for maximal efficacy benefit. Patients who undergo dose modifications retain clinical benefit, regardless of whether the dose modification occurs early or late in therapy. The ixabepilone every 3-week dosing schedule fulfills public health guidelines to reduce the need for in-person care during periods of social distancing. Combination treatment with chemotherapeutic and targeted agents has been under investigation; however, some combinations have shown poor tolerability in small trials. Future studies on efficacy and safety within special populations may assist in creating individualized treatment plans for mBC patients with hard-to-treat characteristics, as well as in other tumor types subject to chemotherapy resistance, such as platinum-resistant ovarian cancer. Clinical trials are needed to evaluate the safety and efficacy of alternative dosages or treatment schedules for improved tolerability. With a distinct mechanism of resistance from other antitumor agents used in mBC management, ixabepilone in combination with capecitabine or as monotherapy should be considered for patients undergoing sequential therapy for mBC.

## Author Contributions

The author confirms being the sole contributor of this work and has approved it for publication.

## Funding

Funding was provided by R-Pharm US. The funder was not involved in the study design, collection, analysis, interpretation of data, the writing of this article or the decision to submit it for publication.

## Conflict of Interest

The author declares that the research was conducted in the absence of any commercial or financial relationships that could be construed as a potential conflict of interest.
